# Study on the prediction effect of a combined model of SARIMA and LSTM based on SSA for influenza in Shanxi Province, China

**DOI:** 10.1186/s12879-023-08025-1

**Published:** 2023-02-06

**Authors:** Zhiyang Zhao, Mengmeng Zhai, Guohua Li, Xuefen Gao, Wenzhu Song, Xuchun Wang, Hao Ren, Yu Cui, Yuchao Qiao, Jiahui Ren, Limin Chen, Lixia Qiu

**Affiliations:** 1 Department of Health Statistics, School of Public Health, Shanxi Medical University, Taiyuan, Shanxi China; 2 Shanxi Centre for Disease Control and Prevention, Taiyuan, 030012 Shanxi China; 3 Shanxi Provincial Peoples Hospital, Taiyuan, Shanxi China

**Keywords:** Influenza, SARIMA model, LSTM model, SSA, Predicting

## Abstract

**Background:**

Influenza is an acute respiratory infectious disease that is highly infectious and seriously damages human health. Reasonable prediction is of great significance to control the epidemic of influenza.

**Methods:**

Our Influenza data were extracted from Shanxi Provincial Center for Disease Control and Prevention. Seasonal-trend decomposition using Loess (STL) was adopted to analyze the season characteristics of the influenza in Shanxi Province, China, from the 1st week in 2010 to the 52nd week in 2019. To handle the insufficient prediction performance of the seasonal autoregressive integrated moving average (SARIMA) model in predicting the nonlinear parts and the poor accuracy of directly predicting the original sequence, this study established the SARIMA model, the combination model of SARIMA and Long-Short Term Memory neural network (SARIMA-LSTM) and the combination model of SARIMA-LSTM based on Singular spectrum analysis (SSA-SARIMA-LSTM) to make predictions and identify the best model. Additionally, the Mean Squared Error (MSE), Mean Absolute Error (MAE) and Root Mean Squared Error (RMSE) were used to evaluate the performance of the models.

**Results:**

The influenza time series in Shanxi Province from the 1st week in 2010 to the 52nd week in 2019 showed a year-by-year decrease with obvious seasonal characteristics. The peak period of the disease mainly concentrated from the end of the year to the beginning of the next year. The best fitting and prediction performance was the SSA-SARIMA-LSTM model. Compared with the SARIMA model, the MSE, MAE and RMSE of the SSA-SARIMA-LSTM model decreased by 38.12, 17.39 and 21.34%, respectively, in fitting performance; the MSE, MAE and RMSE decreased by 42.41, 18.69 and 24.11%, respectively, in prediction performances. Furthermore, compared with the SARIMA-LSTM model, the MSE, MAE and RMSE of the SSA-SARIMA-LSTM model decreased by 28.26, 14.61 and 15.30%, respectively, in fitting performance; the MSE, MAE and RMSE decreased by 36.99, 7.22 and 20.62%, respectively, in prediction performances.

**Conclusions:**

The fitting and prediction performances of the SSA-SARIMA-LSTM model were better than those of the SARIMA and the SARIMA-LSTM models. Generally speaking, we can apply the SSA-SARIMA-LSTM model to the prediction of influenza, and offer a leg-up for public policy.

## Background

Influenza, whose incidence often ranks first among notifiable infectious diseases, is an acute respiratory infectious disease caused by the influenza virus, seriously damaging human health [1]. The main clinical manifestations comprise acute high fever, physical pain, and fatigue and are accompanied by respiratory symptoms such as cough or sore throat. However, some special groups, such as infants, pregnant women, the elderly and those with chronic basic diseases are prone to complications and even death. Since the influenza virus is easy to mutate and features easy infection, transmission and diffusion, which leads to outbreaks and epidemics, the occurrence and development of influenza often causes major public health problems [2]. In recent years, global integration has greatly promoted the mobility of groups, which increases the risk of influenza pandemics [3]. Accurate and reasonable prediction provides reliable information and basis for prevention and control, which can enable people to detect abnormal trends in time and contain the epidemic at an early stage, thus reducing human health hazards and economic burdens [4].

In the past, linear models such as the grey prediction model [5], the exponential smoothing method [6], the autoregressive integrated moving average (ARIMA) model and the SARIMA model [7] were often used to predict infectious diseases. The SARIMA model, one of the classical prediction models of infectious diseases, is often used as a benchmark to evaluate many new modelling methods [8]. In 2021, Song used a SARIMA model to predict the incidence of influenza-like illness in high-risk regions in the United States from 2011 to 2020. The results showed that the SARIMA model was suitable for forecasting the ILI incidence of Mississippi [9]. However, these models are constructed by linear information, which show certain limitations in the nonlinear part [10]. Moreover, SARIMA model needs to make the unstable sequence stationary by difference, which will lose certain information and reduce the accuracy of prediction. Therefore, nonlinear models based on machine learning theory, such as Support Vector Machines (SVM) [11], Multivariate Adaptive Regression Splines (MARS) [12], Random Forest (RF) [13] and Recurrent Neural Networks (RNN) [14], are widely used in the field of time series prediction. In 2022, Dai used a hybrid model combining XGBoost, four GARCH models and MLP model (XGBoost-GARCH-MLP) to predict PM concentration values and volatility. The results showed that the combined model based on machine learning was more accurate in predicting PM values [15]. Compared with SARIMA model, RNN has strong nonlinear, mapping and adaptive characteristics, which can effectively improve the prediction accuracy [14]. Moreover, compared with other machine learning models, RNN has a deeper hidden layer and learning ability, which not only ensures the ability to express the nonlinearity of time series, but also considers the time correlation. However, when the time series is long, RNN will suffer from gradient explosion and lack of long-term memory [8]. Long-Short Term Memory Neural Networks (LSTM) introduces a unique memory unit structure, which can make up for the deficiency of RNN and is more suitable for processing long time series data [16]. This property makes it one of the most powerful tools for predicting nonlinear time series in practical applications. In 2017, Li used SVM, Naive Bayes, Decision Tree, Multiple-layer Perceptron, RNN and LSTM to predict the stock data of China from 2008 to 2015. The results showed that Multiple-layer Perceptron, RNN and LSTM were better [17]. In recent years, LSTM and other neural network models have been gradually applied in the field of public health. In 2022, Zhu established a LSTM model to predict the incidence of influenza in Fujian Province, China from 2019 to 2021. The results showed that LSTM had good predictive performance [18]. In 2021, Dai established a deep learning model for an atmospheric pollutant memory network (LSTM) by both applying the one-dimensional multi-scale convolution kernel (ODMSCNN) and a LSTM on the basis of temporal and spatial characteristics. The results showed that the air pollutant concentration prediction model based on ODMSCNN-LSTM had a better prediction effect compared with multi-layer perceptron (MLP), CNN, and LSTM models [19].

Time series are usually considered to consist of both linear and nonlinear components [20]. Neither linear model nor nonlinear model can fully capture all the information of time series. Based on this, many scholars have proposed combination models composed of linear and nonlinear models. In 2016, Oliveira proposed the ARIMA-SVR combination model, and the results proved that the hybrid model can effectively improve the prediction accuracy [21]. In 2021, Zhai used the combination model of ARIMA-ERNN and ARIMA-BPNN to predict brucellosis in Shanxi Province, China. The results showed that combination models were better than the single ARIMA model [10]. Nevertheless, the above models all predicted the original series. When the characteristics of the original series are complex, the accuracy of using the combined model directly to predict the original sequence is still insufficient [22].

To solve this problem, this research proposed a combination model construction strategy based on decomposition and recombination. Singular spectrum analysis (SSA) can decompose the complex original sequence into some simple and regular sub-sequences [23]. The prediction model can be indirectly established by modeling and superimposing the sub-sequences, which can improve the prediction accuracy of the model. In recent years, the prediction model based on SSA has been gradually applied to public health, stock price prediction and mechanical engineering. In 2021, Mahdi used the SSA method to analyze and predict COVID-19. The results showed that the combined prediction model enjoyed significantly higher accuracy than the single model [24].

In 2019, Xiao et al. used SSA to decompose and reconstruct the stock price and forecast it. The results showed that the performance of the combined prediction model was better than the single prediction method [25]. In 2019, Zhang et al. used SSA to decompose and reconstruct the short-term wind power, and modeled and predicted the decomposition sequence respectively to improve the prediction accuracy [26].

In this study, the SARIMA model, SARIMA-LSTM model and SSA-SARIMA-LSTM model were established based on the weekly Influenza-Like Illness (ILI) patient ratio from 2010 to 2018 in Shanxi Province to evaluate the fitting effect of the three models. Three models were used to predict the 2019 influenza data, respectively, to evaluate the prediction performance of the three models.

The innovation of this paper lies in the establishment of the indirect combination prediction model SSA-SARIMA-LSTM through the idea of decomposition and combination. Compared with single prediction model and direct modeling prediction, SSA-SARIMA-LSTM is more accurate in predicting influenza. This study, more targeted in prevention, and more reasonable in medical resource allocation, will provide more effective theoretical support for the prevention and control of influenza in Shanxi Province, China to effectively reduce the health hazards and economic burden caused by influenza.

## Methods

### Data sources

In this study, a total of 520 weeks of influenza data from the 1st week of 2010 to the 52nd week of 2019 were obtained from Shanxi Provincial Center for Disease Control and Prevention, China. All cases were diagnosed under the ‘Diagnostic criteria for influenza (WS 285-2008)’ [27]. Influenza data include the number of ILI patients and the total number of outpatient and emergency cases in the same period. To eliminate the difference [28], ILI patient ratio was calculated as

1 $$ILI{\text{\% }} = \frac{the \, number \, of \, ILI \, patients}{{the \, total \, number \, of \, outpatient \, and \, emergency \, cases}}$$

The influenza cases from the 1st week in 2010 to the 52nd week in 2019 were assembled as weekly counts. The weekly ILI% from the 1st week in 2010 to the 52nd week in 2018 were used to build the SARIMA model. The fitted data of the SARIMA model were taken as the input of neural networks, which were divided into two sections: a training set and a verification set. The data from the 1st week of 2010 to the 52nd week of 2017 were used as the training set to construct the neural network, and the data from the 1st week of 2018 to the 52nd week of 2018 were used as the verification set to validate the neural network. The weekly ILI% from the 1st week to the 52nd week of 2019 were used as the test set to test the prediction performance of the three models.

### Analysis of influenza sequence characteristics

STL [29] can be used to analyze the long-term trend, seasonal trend and random effect of influenza in Shanxi Province from the 1st week of 2010 to the 52nd week of 2019 as follows:

2 $$Xt = Tt + St + It$$

where *X*_*t*_ is the actual value of ILI% at time t and *T*_*t*_, *S*_*t*_ and *I*_*t*_ are the long-term trends, seasonal trends and random effects, respectively.

### SARIMA model

SARIMA, a classic model in many time series analyses, is usually constructed as SARIMA (p, d, q) (P, D, Q) _s_ as follows [30]:

3 $$\Theta_{P} (B^{S} )\theta_{p} (B)(1 - B^{S} )^{D} (1 - B)^{d} x_{t} = \Phi_{Q} (B^{S} )\phi_{q} (B)w_{t}$$

where $\Theta_{P}$, $\theta_{p}$, $\Phi_{Q}$ and $\phi_{q}$ are polynomials of order P, order p, order Q and order q, respectively. p and q represent the order of autoregressive and moving average. d and D represent the order of trend differencing and seasonal differencing. P, Q and s represent the order of seasonal autoregressive, seasonal moving average and seasonal periodicity, respectively. In this study, the weekly ILI% from the 1st week in 2010 to the 52nd week in 2018 were used to build the SARIMA model, and the process included the following steps. First, the stationarity of the sequence was checked by using the Augmented Dickey-fuller (ADF) test, the Kwiatkowski-Phillips-Schmidt-Shin (KPSS) test and the autocorrelation function (ACF) plot. If the *p* value of the ADF test was less than the significance level, and the autocorrelation coefficient decayed rapidly to 0, the sequence was considered to be stationary. If the *p* value of the KPSS test was less than the significance level, the sequence was considered to be non-stationary. The Ljung-Box test was used to test whether the sequence was the white noise sequence, and if the *p* value was less than the significance level, the sequence had no randomness. Second, when the original sequence was stationary and non-random, the model can be directly constructed. When the original sequence was not stable, d or D-order difference was used to make the sequence stable and then constructed the model. Afterwards, Python Grid Search was used to automatically fit the SARIMA model. According to the minimum Akaike information criterion (AIC), the optimal model was selected, and the success of model fitting was judged by the residual white noise test. Maximum likelihood estimation (MLE) was used to perform the parameter test of the model [8]. Finally, the data from the 1st week to the 52nd week of 2019 were predicted by this model, and the prediction effect of the model was tested.

### LSTM model

**Fig. 1:**
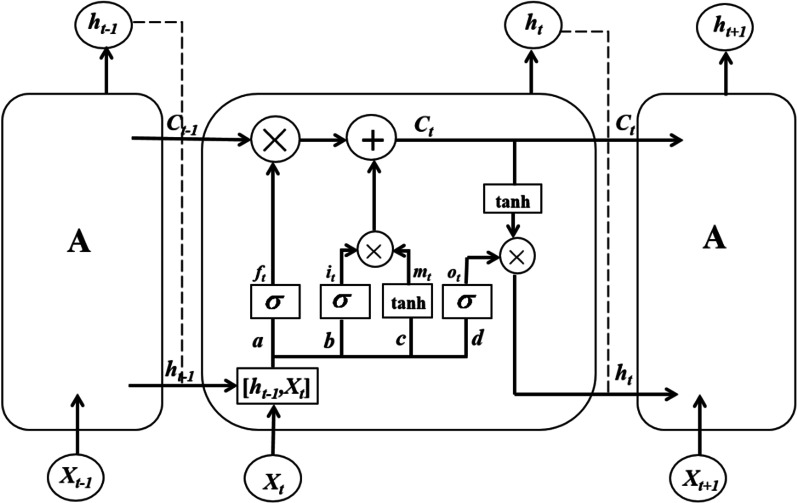
The LSTM unit: A is Memory module, a is the forgetting gate, b and c are the input gate, d is the output gate

LSTM hidden layer module, also known as Memory module (A), was shown in Fig. 1. It consists of a cell and three gates: INPUT GATES, FORGET GATES, and OUTPUT GATES [16].

The mathematical formulas of LSTM used in this study are shown as follows [31]. By receiving the output value *h*_*t-1*_ of the previous state and the input value *X*_*t*_ of the current moment, the forgetting gate uses the sigmoid function to determine the retention degree *f*_*t*_ of the transmitted information.

4 $$f_{t} = \sigma \left( {W_{fh} h_{t - 1} + W_{fx} x_{t} + b_{f} } \right)$$

The input gate updates the current state *C*_*t*_ by using sigmoid and Tanh functions to pass to the next memory cell.

5 $$i_{t} = \sigma \left( {W_{ih} h_{t - 1} + W_{ix} x_{t} + b_{i} } \right)$$

6 $$\mathop {C_{t} }\limits^{ \wedge } = \tanh \left( {W_{ch} h_{t - 1} + W_{cx} x_{t} + b_{c} } \right)$$

7 $$C_{t} = f_{t} C_{t - 1} + i_{t} \mathop {C_{t} }\limits^{ \wedge }$$

The output gate outputs the value *h*_*t*_ at the current time.

8 $$o_{t} = \sigma \left( {W_{oh} h_{t - 1} + W_{ox} x_{t} + b_{o} } \right)$$

9 $$h_{t} = o_{t} \tanh \left( {C_{t} } \right)$$

The model controls the flow of information in memory units and neural networks through the gates. *W* is the weight matrix. *b* is the bias term. $\sigma$ is the sigmoid function. In this paper, LSTM was set to 1000 iterations, batch size was set to 256, the learning rate was 0.001, time step was set to 1, the number of hidden layers was 1, Adam algorithm was adopted to optimize parameters, and other parameters were default. The value range of hidden layer neurons were calculated by the following empirical formula, where *m* and *n* are the number of neurons in the input layer and output layer respectively, which we set to 1, and *k* is a constant between 1 and 10:

10 $$M = \sqrt {m + n} + k$$

### SARIMA-LSTM model and prediction process of influenza

**Fig. 2:**
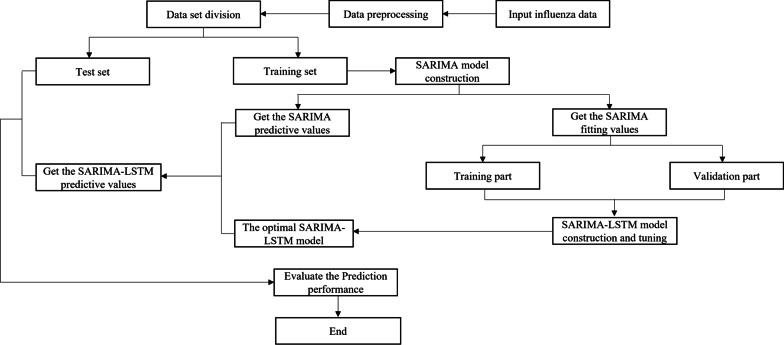
Flow chart of influenza SARIMA-LSTM prediction model

The SARIMA model is suitable for extracting the linear part of the original time series, but it shows certain limitations in the nonlinear part [20]. The LSTM model has the characteristics of strong nonlinearity, mapping and adaptability, which can reduce the error of the SARIMA model. Therefore, the combined model of SARIMA-LSTM was constructed in this study, which can comprehensively improve the prediction accuracy of the model. Figure 2 showed the prediction process of influenza and the construction framework of SARIMA-LSTM model, including four parts: data pre-processing, SARIMA model construction, SARIMA-LSTM model tuning and data prediction.

1. Data processing preparation. Data preprocessing was performed in the original data of influenza, and the data set was divided into training set and test set. The training set, from the 1st week of 2010 to the 52nd week of 2018, was used to construct the optimal SARIMA model. The test set was used to verify the performance of the model.
2. SARIMA model construction. Input the training set into SARIMA to build the optimal SARIMA model. The fitting value was obtained, and the error was calculated by the following formula:
  11 $${\text{e}}_{t} = y_{t} - \mathop {L_{t} }\limits^{ \wedge }$$
  where $y_{t}$ is the actual value of the original series, $\mathop {L_{t} }\limits^{ \wedge }$ is the fitting value of the optimal SARIMA, and ${\text{e}}_{t}$ is the error, also known as the residual. The ILI% data of the first 53 weeks were lost in this step due to a first-order difference and a seasonal difference in the construction of the optimal SARIMA model. The established SARIMA model was used to obtain the fitting values from the 2nd week of 2011 to the 52nd week of 2018. The data from the 2nd week of 2011 to the 52nd week of 2017 were used as the training part of the LSTM model, and the data from the 1st week of 2018 to the 52nd week of 2018 were used as the validation part of the LSTM model.
3. SARIMA-LSTM model tuning. The training part was used as the input, and ILI% at the same time point was used as the output to construct the SARIMA-LSTM model, and the verification part was used to optimize the model. In order to improve model training speed and prediction accuracy, the Min–Max normalization method [30] was used to normalize the original data.
  12 $$X^{*} = \frac{{X - X_{\min } }}{{X_{\max } - X_{\min } }}$$
  X^*^ is the normalized value of the data, *X* is the original data, *X*_*max*_ and *X*_*min*_ are the maximum and minimum values respectively. Since hidden layer nodes have a great impact on the performance of the model, we chose MSE, MAE and RMSE as the evaluation indexes of network performance. Through experiments, the hidden layer neurons were selected when the smallest MSE, MAE and RMSE to construct the optimal SARIMA-LSTM model.
4. Data prediction. The established SARIMA model was used to predict influenza data from the 1st week to the 52nd week in 2019, and the predicted values were used as the input values of the SARIMA-LSTM model to obtain the output values. The inverse normalization method was used to restore the output values into meaningful data. The predicted values were compared with the real values of the test set to evaluate the Prediction performance of the model.

### Singular spectrum analysis

Singular spectrum analysis, proposed by Broomhead and King in 1986, has been widely used in the field of time series decomposition in recent years. By transforming the original sequence into a trajectory matrix for decomposition and reconstruction, SSA can decompose it into the long-term trend, periodic trend and noise, to further forecast. The specific decomposition process is as follows:

1. Embedding. In this paper, the original sequence *X* = *(X*_*1*_*, X*_*2*_*,…, X*_*N*_*)* was transformed into a sequence of *K* vectors with length *L(2* ≤ *L* ≤ *2/N)*. *L* is an integer value called window length and *K* is an integer such that the trajectory matrix includes all values, *K* = *N-L* + 1. When the time sequence data has obvious periodic characteristics, the window length is set to an integer multiple of the period which is less than one-third of the total length [23].
  13 $$X = \left[ {\begin{array}{*{20}c} {X_{1} } & {X_{2} } & \cdots & {X_{K} } \\ {X_{2} } & {X_{3} } & \cdots & {X_{K + 1} } \\ \vdots & \vdots & {} & \vdots \\ {X_{L} } & {X_{L + 1} } & \cdots & {X_{N} } \\ \end{array} } \right]$$
2. Singular value decomposition. Let $S = XX^{T}$,$U_{1} ,U_{2} , \ldots ,U_{L}$ be the eigenvectors of *S*, and $\lambda_{1} \ge \lambda_{2} \ge \cdots \ge \lambda_{L}$, its corresponding eigenvalues. Let $V_{i} = X^{T} U_{i} /\sqrt {\lambda_{i} }$,$U_{i}$ and $V_{i}$ be the left and right singular vectors of matrix *X* respectively, and $\sqrt {\lambda_{i} } (i = 1,2, \ldots L)$, its corresponding singular values. At this time, *X* can be expressed as *X* = *E*_*1*_ + *E*_*2*_ + *…* + *E*_*L*_, and $E_{i} = \sqrt {\lambda_{i} } U_{i} V_{i}^{T} \left( {i = 1,2, \ldots ,L} \right)$.
3. Grouping and diagonal averaging. We divided *X* into *r* disjoint subsets according to the contribution rate of singular values ($X = E_{{I_{1} }} + E_{{I_{2} }} + \ldots + E_{{I_{{\text{p}}} }}$). Then, using anti-diagonal averaging, we transformed the new trajectory matrix ($E_{{I_{1} }} ,E_{{I_{2} }} , \ldots ,E_{{I_{p} }}$) into new sequences of length *N* and total number *p*. Finally, the original sequence *X* was decomposed into *p* subsequences with length *N*, and the sum of subsequences was *X*.
  14 $$e_{k} = \left\{ {\begin{array}{*{20}c} {\frac{1}{k}\sum\limits_{p = 1}^{k} {e_{p,k - p + 1}^{ * } } } & {{\text{for }}(1 \le k \le m^{ * } )} \\ \begin{gathered} \frac{1}{{m^{ * } }}\sum\limits_{p = 1}^{{m^{ * } }} {e_{p,k - p + 1}^{ * } } \hfill \\ \frac{1}{N - k + 1}\sum\limits_{{p = k - n^{ * } + 1}}^{{N - n^{ * } + 1}} {e_{p,k - p + 1}^{ * } } \hfill \\ \end{gathered} & \begin{gathered} {\text{for }}(m^{ * } \le k \le n^{ * } ) \hfill \\ {\text{for }}(n^{ * } \le k \le N) \hfill \\ \end{gathered} \\ \end{array} } \right.$$
  *E* is a matrix*(m* × *n)*, $m^{*} = \min \left\{ {m,n} \right\}$,$n^{*} = \max \left\{ {m,n} \right\}$,*N* is the total number of inverse diagonals *N* = *m* + *n-1*, *k* = *1,2,…,N*. According to the above formulas, *E* is transformed into a one-dimensional time series $e_{1} ,e_{2} , \ldots ,e_{N}$ [32].

### SSA-SARIMA-LSTM model and prediction process of influenza

**Fig. 3:**
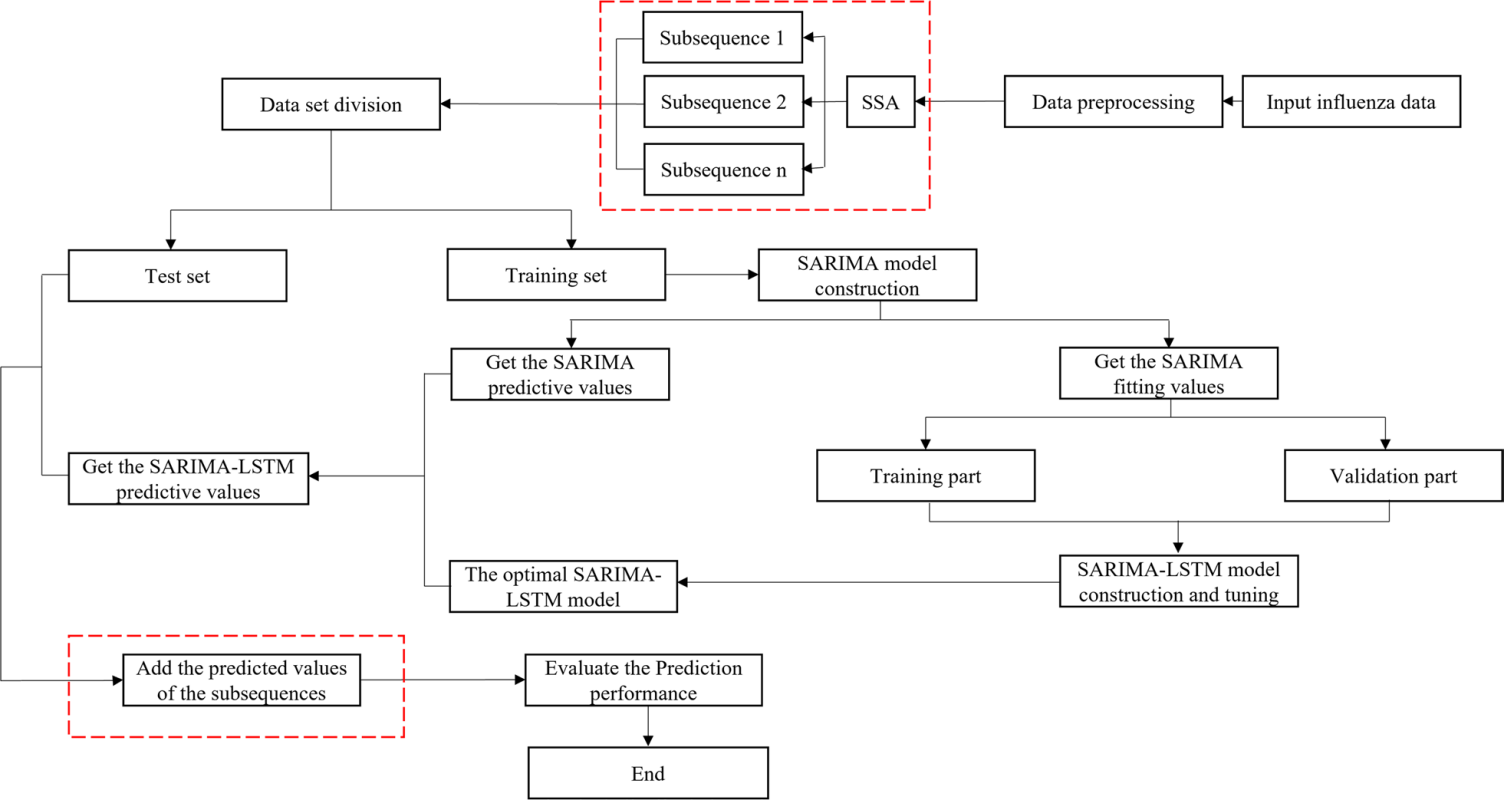
Flow chart of influenza SSA-SARIMA-LSTM prediction model. The sequence decomposition step and the sequence combination step are surrounded by dashed lines

It is difficult for a single model to capture the comprehensive characteristics of signals for accurate prediction. Therefore, we proposed an indirect prediction method based on SSA. Compared with the SARIMA-LSTM model, the construction of the SSA-SARIMA-LSTM model added the sequence decomposition step and the sequence combination step (Fig. 3).

1. Data processing preparation and decomposition. First, we used SSA to transform the original sequence from the 1st week of 2010 to the 52nd week of 2018 into multiple simple subsequences. The partitioning process of data sets was consistent with the SARMI-LSTM model.
2. SARIMA-LSTM model building. Second, we constructed the SARIMA-LSTM model for each subsequence.
3. Data prediction. Third, the optimal SARIMA model prediction values of each subsequence from the 1st week to the 52nd week in 2019 were taken as the input values of the model to obtain the output prediction values, and the inverse normalization method was used to restore the output prediction values of the subseries to meaningful data.
4. Sequence combination. Finally, the predicted values of each subsequence were added to obtain the predicted values of the SSA-SARIMA-LSTM model from the 1st week to the 52nd week in 2019. The predicted values were compared with the real values of the test set to evaluate the Prediction performance of the model.

### Indicators of model performance

Three performance indexes, MSE, MAE and RMSE, were used to assess the fitting and prediction effects of those models.

15 $$MSE = \frac{1}{N}\sum\limits_{k = 1}^{N} {\left( {X_{k} - \mathop {X_{k} }\limits^{ \wedge } } \right)^{2} }$$

16 $$MAE = \frac{1}{N}\sum\limits_{k = 1}^{N} {\left| {X_{k} - \mathop {X_{k} }\limits^{ \wedge } } \right|}$$

17 $$RMSE = \sqrt {\frac{1}{N}\sum\limits_{k = 1}^{N} {(X_{k} - \mathop {X_{k} }\limits^{ \wedge } )^{2} } }$$

$X_{{\text{k}}}$ is the actual value at time *k*. $\mathop {X_{{\text{k}}} }\limits^{ \wedge }$ is the predicted value of the model. *N* is the sample size.

### Data analysis

Excel software version 2021 was used for data collection and collation, Anaconda software version 4.10.3 was used to establish STL, the SARIMA model, the SARIMA-LSTM combined model and the SSA-SARIMA-LSTM combined model. MATLAB software version 2019 was used for SSA.

## Results

### Seasonal characteristics of influenza

**Fig. 4:**
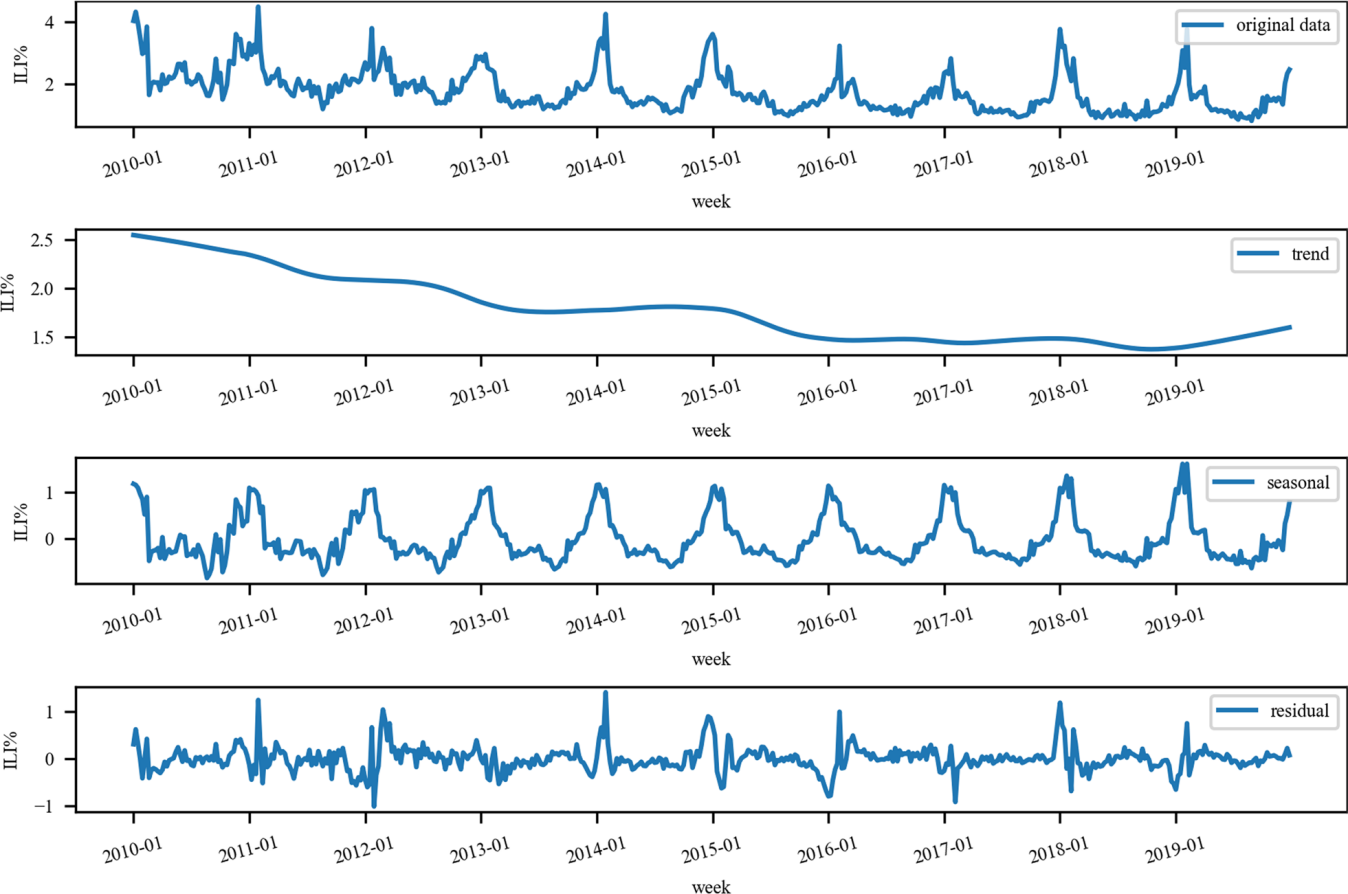
Seasonal decomposition based on STL of the influenza in Shanxi Province from 2010 to 2019

STL was used to study the time series of ILI% in Shanxi Province from the 1st week of 2010 to the 52nd week of 2019, and the results were shown in Fig. 4. The original data, long-term trends, seasonal trends and residuals were shown from top to bottom. Based on the long-term trends, Influenza in Shanxi Province decreased year by year. The seasonal trends revealed that the Influenza in Shanxi Province showed obvious seasonality and periodicity, with a cycle of 1 year (52 weeks). In a cycle, the peak of influenza in Shanxi Province, China was mainly at the beginning and end of the year.

### SARIMA model

**Fig. 5:**
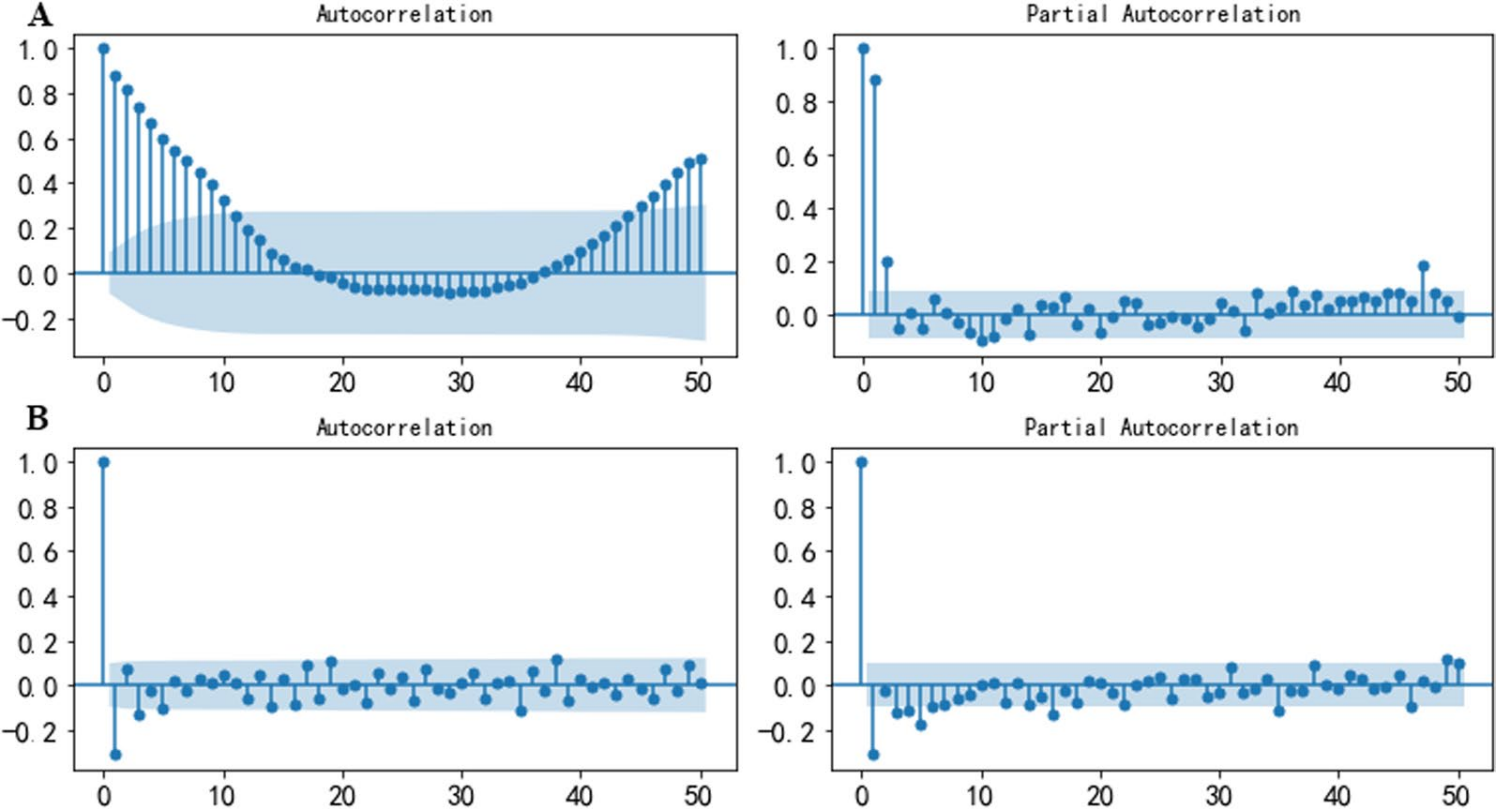
Autocorrelation and partial autocorrelation plots: **A** is the original time series, and **B** is the adjusted time series

Weekly ILI% from the 1st week in 2010 to the 52nd week in 2018 in Shanxi Province were used to build the SARIMA model. The ACF of the original series showed obvious seasonal characteristics (Fig. 5). The ADF test: *t* = − 5.249, *P* < 0.001, and the KPSS test: *χ*^*2*^ = 1.251, *P* = 0.010, and the Ljung-Box test: *χ*^*2*^ = 352.724, *P* < 0.001. Therefore, the original sequence was non-stationary and non-random. The original series became stationary after the first-order difference and a seasonal difference, and the adjusted sequence was not a random effect (Table 1). Finally, SARIMA (p,1,q) (P,1,Q) _52_ was preliminarily selected.

**Table 1:** ADF, KPSS and Ljung-Box tests of the time series

Time series	ADF		KPSS		Ljung-Box	
	*t*	*P*	*χ*^*2*^	*P*	*χ*^*2*^	*P*
Original series	− 5.249	< 0.001	1.251	0.010	352.724	< 0.001
Adjusted series	− 7.999	< 0.001	0.098	0.100	42.375	< 0.001

By using Python grid to search the minimum AIC, we finally determined the SARIMA (2,1,1) (2,1,0) _52_ model (AIC = 95.163). The parameters of the SARIMA (2,1,1) (2,1,0) _52_ model were statistically significant and that the residual sequence of the model was a random sequence (*χ*^*2*^ = 0.020, *P* = 0.900) (Table 2).

**Table 2:** Model parameter estimation

Model	Parameter estimate					Fitting index	
	*AR1*	*AR2*	*MA1*	*SAR1*	*SAR2*	*AIC*	*BIC*
(2,1,1)(2,1,0)_52_	0.577*	0.142*	− 0.989*	− 0.700*	− 0.424*	95.163	117.563

*P ≤ 0.05, the same below

### SARIMA-LSTM model

The fitted values of the SARIMA (2,1,1) (2,1,0) _52_ model from the 2nd week of 2011 to the 52nd week of 2017 were used as inputs, and the actual values at the same time were used as outputs to establish the SARIMA-LSTM model. The data from weeks 1 through 52 of 2018 were used to verify the neural network. According to the formula (10), the hidden layer neurons of the SARIMA-LSTM were between 2 and 12. Through experiments, when the hidden layer neuron was 4, the MSE, MAE and RMSE of the verification set reached the minimum; that is, the number of hidden layers of the model was set to 1 and the nodes were set to 4, (Table 3). Finally, the predicted values of the SARIMA (2,1,1) (2,1,0) _52_ model from weeks 1 through 52 of 2019 were used as the inputs. The established SARIMA-LSTM model was used to obtain the output predicted values, and the inverse normalization was performed.

**Table 3:** Validation set error of the SARIMA-LSTM model

Neuron number	MSE	MAE	RMSE
2	0.0513	0.1602	0.2264
3	0.0521	0.1601	0.2282
**4**	**0.0512**	**0.1578**	**0.2264**
5	0.0516	0.1602	0.2271
6	0.0518	0.1601	0.2275
7	0.0516	0.1612	0.2272
8	0.0513	0.1594	0.2266
9	0.0514	0.1592	0.2268
10	0.0523	0.1595	0.2287
11	0.0515	0.1588	0.2268
12	0.0516	0.1605	0.2271

Bold values indicate the optimal hidden layer neuron

### SSA-SARIMA-LSTM model

The original influenza sequence in Shanxi Province was complex, and the accuracy of direct prediction was insufficient. In this study, SSA was used to decompose the ILI% of 468 weeks in Shanxi Province from the 1st week of 2010 to the 52nd week of 2018, and multiple simple and regular subsequences were obtained. The SSA-SARIMA-LSTM model was obtained by building the SARIMA-LSTM model for the subsequences.

#### SSA

**Fig. 6:**
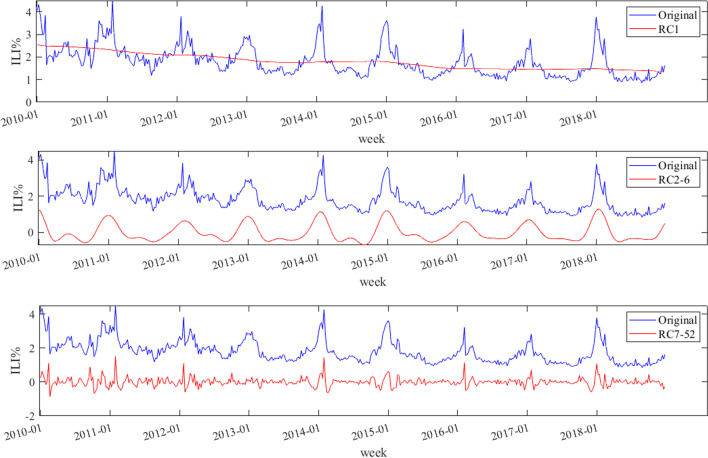
SSA reconstruction

*L* (window length) and *r* (reconstruction number) should be determined, before the SSA decomposition. First,* L* was set to 52 due to the cyclical nature of influenza. Afterwards, we obtained 52 singular values which were from large to small by using SSA. The contribution rate of the first singular value was the largest (90.86%), 2–6 followed (6.93%), and the contribution rate of 7–52 was the smallest (2.18%). The matrices corresponding to the singular values of 1, 2–6 and 7–52 were grouped and reconstructed into three subsequences RC_1_, RC_2-6_ and RC_7-52_ by diagonal averaging. The three subsequences showed different characteristics: RC_1_ showed a gradual downward trend, RC_2-6_ showed periodic fluctuation, and RC_7-52_ fluctuated around the mean with no obvious trend (Fig. 6).

#### Constructing SSA-SARIMA-LSTM model

SSA was used to reconstruct the original influenza sequence into three subsequences with different periodicity and stability. Afterwards, we tested the stationarity and white noise of each subsequence. The three original subsequences were non-random. RC_1_ became stationary after the second-order difference, and RC_2–6_ became stationary after the seasonal difference. RC_7-52_ was a stationary sequence. By using Python grid to search the minimum AIC, SARIMA (2,2,0) (0,0,0)_52_, SARIMA (2,0,2) (0,1,0)_52_ and SARIMA (2,0,2) (2,0,0)_52_ were used to fit RC_1_, RC_2–6_ and RC_7–52_, respectively (Table 4). Consistent with the construction method of the SARIMA-LSTM model, the number of hidden layers of the models was set to 1 and the hidden layer neurons of RC_1_, RC_2–6_ and RC_7–52_ sequences were 11, 11 and 7, respectively (Table 5). The SARIMA-LSTM models were used to predict the ILI% of subsequences from weeks 1 through 52 of 2019, and the predicted values of subsequences were added to obtain the predicted values of the SSA-SARIMA-LSTM model.

**Table 4:** Model parameter estimation of the subsequences

Model	Parameter estimate							Fitting index
	*AR1*	*AR2*	*MA1*	*MA2*	*SAR1*	*SAR2*	*AIC*	*BIC*
SARIMA (2,2,0) (0,0,0)_52_	0.290*	− 0.035*	–	–	–	–	− 5640.532	− 5628.112
SARIMA (2,0,2) (0,1,0)_52_	1.936*	− 0.976*	0.878*	0.732*	–	–	− 3091.174	− 3071.056
SARIMA (2,0,2) (2,0,0)_52_	1.710*	− 0.896*	− 1.983	1.000	− 0.144*	− 0.054	− 201.267	− 174.026

**Table 5:** Validation set error of the subsequences

RC_1_	MSE	MAE	RMSE	RC_2-6_	MSE	MAE	RMSE	RC_7-52_	MSE	MAE	RMSE
2	0.000033	0.004799	0.005719	2	0.009302	0.069116	0.096446	2	0.047598	0.150445	0.218170
3	0.000028	0.004384	0.005261	3	0.008871	0.067164	0.094187	3	0.045702	0.149534	0.213780
4	0.000016	0.003391	0.004022	4	0.008510	0.065193	0.092250	4	0.045326	0.149593	0.212898
5	0.000013	0.003072	0.003586	5	0.008616	0.065786	0.092822	5	0.045352	0.149477	0.212960
6	0.000013	0.003034	0.003539	6	0.008441	0.064807	0.091877	6	0.045534	0.149527	0.213387
7	0.000019	0.003733	0.004411	7	0.008822	0.066768	0.093925	**7**	**0.045288**	**0.149446**	**0.212810**
8	0.000009	0.002576	0.003012	8	0.008425	0.064574	0.091785	8	0.045329	0.149643	0.212906
9	0.000009	0.002523	0.002983	9	0.008418	0.064436	0.091748	9	0.045363	0.149636	0.212985
10	0.000007	0.002193	0.002593	10	0.008425	0.064592	0.091790	10	0.045361	0.149782	0.212982
**11**	**0.000006**	**0.002130**	**0.002409**	**11**	**0.008388**	**0.064291**	**0.091585**	11	0.045420	0.149730	0.213120
12	0.000007	0.002161	0.002570	12	0.008393	0.064421	0.091611	12	0.045423	0.149752	0.213126

Bold values indicate the optimal hidden layer neuron

#### Comparison of the three models

**Fig. 7:**
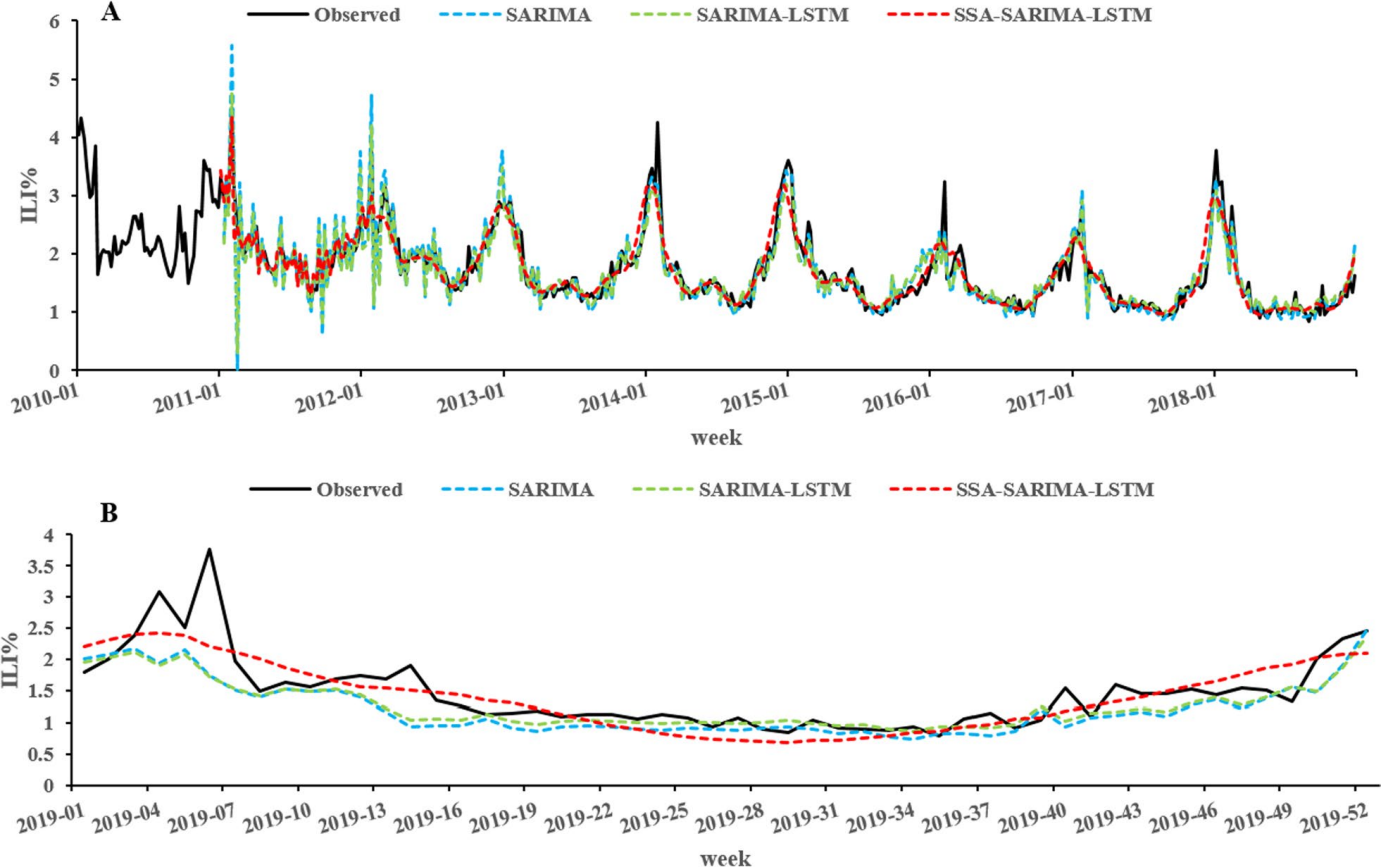
The fitted and predicted values of the three models. A was the fitting part, and B was the prediction part

The SARIMA model, the SARIMA-LSTM model and the SSA-SARIMA-LSTM model were used to predict the ILI% in Shanxi Province from the 1st week of 2019 to the 52nd week. The predicted and fit values of the three models and the ILI% were shown in Fig. 7. To objectively evaluate the model performance, the fitting and prediction performances of the three models were compared by MSE, MAE and RMSE (Table 6). Compared with the SARIMA model, the MSE, MAE and RMSE of the SARIMA-LSTM model decreased by 13.75, 3.26 and 7.13%, respectively, in fitting performance; the MSE, MAE and RMSE decreased by 8.60, 12.36 and 4.39%, respectively, in prediction performances. Compared with the SARIMA model, the MSE, MAE and RMSE of the SSA-SARIMA-LSTM model decreased by 38.12, 17.39 and 21.34%, respectively, in fitting performance; the MSE, MAE and RMSE decreased by 42.41, 18.69 and 24.11%, respectively, in prediction performances. Compared with the SARIMA-LSTM model, the MSE, MAE and RMSE of the SSA-SARIMA-LSTM model decreased by 28.26, 14.61 and 15.30%, respectively, in fitting performance; the MSE, MAE and RMSE decreased by 36.99, 7.22 and 20.62%, respectively, in prediction performance.

**Table 6:** Comparison of the three models in fitting and prediction performances

Model	Fitting performance			Prediction performance		
	MSE	MAE	RMSE	MSE	MAE	RMSE
SARIMA	0.085	0.183	0.291	0.187	0.283	0.432
SARIMA-LSTM	0.073	0.177	0.270	0.171	0.248	0.413
**SSA-SARIMA-LSTM**	**0.052**	**0.151**	**0.229**	**0.108**	**0.230**	**0.328**

Bold values indicate the smallest MSE, MAE, RMSE

## Discussion

As the influenza virus is prone to mutation, it is highly vulnerable to an epidemic, even a worldwide pandemic, which will increase the burden on health services and economic losses [33]. It’s the key to preventing and controlling the harm of influenza which requires a timely understanding of the epidemic trend of influenza and early detection of the epidemic situation. ILI% in Shanxi Province showed a downward trend from the 1st week of 2010 to the 52nd week of 2019, and the analysis of Seasonal decomposition based on STL (Fig. 4) showed significant seasonal characteristics. The peak was mainly concentrated at the beginning and end of each year, which was a typical characteristic of the influenza epidemic in Northern China. The main reason may be related to the cold and dry weather in winter. Low temperature makes the virus stay alive longer, and low humidity makes the virus stay in the air longer, both of which increase the susceptibility of influenza to cause a high incidence of influenza [34]. Therefore, it is necessary to strengthen education attainment, raise awareness of prevention and encourage influenza vaccination to avoid the economic and disease burden caused by influenza.

Many factors influence the occurrence of influenza, and it is difficult to collect data on influencing factors. Facing this situation, time series prediction model attributes all the complex external factors to the time factors and predicts the future incidence to overcome the disadvantages of traditional mathematical-statistical methods. The SARIMA model, one of the most classical time series methods in infectious disease prediction, has been proven to have high accuracy, and it’s often used as the evaluation basic for new models [7]. Therefore, we established the optimal model SARIMA (2,1,1) (2,1,0) _52_ as the basic model to evaluate the performance of other models. However, the results showed that the prediction performance of the optimal model SARIMA (2,1,1) (2,1,0) _52_ still had some deficiencies. The possible reason was that influenza, like most infectious diseases, was a combination of linear and nonlinear sequences [10]. The SARIMA model can accurately extract the linear components of the time series, but it has some limitations when dealing with nonlinear information. The LSTM has strong nonlinearity, mapping and self-adaptability, which can effectively improve the prediction accuracy. In this paper, we used influenza data to compare the performances of the SARIMA, SARIMA-LSTM and SSA-SARIMA-LSTM models in fitting and prediction. The study found that compared with the SARIMA model, the MSE, MAE and RMSE of the SARIMA-LSTM and SSA-SARIMA-LSTM models had different degrees of decline in terms of fitting and prediction performances. The possible reason was that the combined models made up for the lack of nonlinear mapping ability of the SARIMA model and improved the prediction performance, consistent with the research results of other scholars [10, 30]. All the above models directly predicted the original series. However, the influenza series were non-stationary, with obvious seasonality, trend and other complex temporal characteristics, and the prediction accuracy of direct modeling was insufficient. Based on this, The SSA was used to decompose the original influenza sequence, and the SARIMA-LSTM model was used to predict the subsequences respectively, and the final predicted values were obtained by superposition to establish the SSA-SARIMA-LSTM model. The results showed that the fitting and prediction performances of the SSA-SARIMA-LSTM model were the best, which may be due to the fact that the original sequence was decomposed into relatively simple, stable and regular subsequences. It was easier for the model to fit the regular subsequences, thus improving the prediction accuracy, which was also consistent with the results of Kalantari [35].

To the best of our knowledge, we are the first one to explore the SSA-SARIMA-LSTM combination model based on SSA for predicting the incidence of Influenza. Its advantage is that the SSA-SARIMA-LSTM model combines the advantages of the SARIMA in linear features and a neuron network in nonlinear features and enhances the capability of a single SARIMA. At the same time, the SSA-SARIMA-LSTM model based on decomposition and recombination can make up for the lack of accuracy of direct use of the original sequence prediction. Second, we selected the optimal SARIMA model by using Python Grid Search to automatically search the minimum AIC, which made the model more accurate and suitable for analyzing influenza data. Third, the use of the SSA-SARIMA-LSTM model helps rationalize the allocation of limited public health resources and the early prevention to control influenza.

However, there are also some limitations. First, the patterns and the incidence of influenza vary from region to region [2]. It needs further study whether the SSA-SARIMA-LSTM model is suitable for other areas. Second, this study only established two combination models, and the superiority of SSA-SARIMA-LSTM model and other models remained to be verified. In the future, the influence factors of influenza will be incorporated into the model and we will compare the SSA-SARIMA-LSTM model with other models. In the future, we plan to use different signal decomposition methods and more neural networks to improve the accuracy of influenza prediction. At the same time, we will use this model to study the predictive performance of influenza in different regions.

## Conclusions

In this study, the time series of influenza in Shanxi Province from 2010 to 2019 showed obvious seasonal characteristics and a trend of decreasing. The fitting and prediction performances of the SSA-SARIMA-LSTM model were better than those of the SARIMA-LSTM and SARIMA models, and the SARIMA-LSTM model was better than the SARIMA model. The SSA-SARIMA-LSTM model was more suitable for predicting the incidence of influenza than the SARIMA-LSTM and SARIMA models.

## Data Availability

The data that support the findings of this study are available from the corresponding author upon reasonable request.
